# Modeling small-angle scattering data of porous and/or bicontinuous structures in *n* dimensions

**DOI:** 10.1107/S1600576726002451

**Published:** 2026-04-30

**Authors:** Henrich Frielinghaus

**Affiliations:** a Forschungszentrum Jülich GmbH, Jülich Centre for Neutron Science JCNS at MLZ, 85748 Garching, Germany; Argonne National Laboratory, USA

**Keywords:** fractal structures, porous structures, microemulsions, small-angle neutron scattering, small-angle X-ray scattering

## Abstract

Fractal structures are often observed in small-angle scattering experiments where a simple power law *q*^−α^ describes the scattering intensity over many orders of magnitude. Most theories, however, level off towards lower *q* in a Guinier scattering that describes a simple maximum size with no specific structure. There is a demand for a scattering model for porous structures with repeated domains of a certain size. This is then observed experimentally as a scattering peak that decays in a power law towards higher *q*. The starting point for the model presented here is the well known Teubner–Strey theory for bicontinuous microemulsions, which is modified to different dimensionalities to match the desired power law. A combination of several such model functions can describe scattering profiles along multiple length scales, similarly to the well known Beaucage model.

## Abstract 2

A small-angle scattering fitting function is derived for porous materials with arbitrary fractal dimension. It includes a correlation peak and a power law at higher *q*.

## 1. Introduction

In the field of small-angle scattering, combining ultra and very small angle neutron or X-ray scattering (USANS, VSANS and USAXS) (Barker *et al.*, 2005 ▸; Magerl *et al.*, 2024 ▸; Ji *et al.*, 2022 ▸; Zhang & Ilavsky, 2010 ▸), or small-angle light scattering (SALS) (Nishida *et al.*, 2008 ▸) and conventional small-angle scattering (SANS, SAXS) (Schelten & Schmatz, 1980 ▸; Pilz *et al.*, 1979 ▸), one obtains a scattering curve often ranging from ∼10^−5^ to ∼1 Å^−1^ that may or may not be extended to the wide-angle range. Those curves often describe power laws over many length scales according to *q*^−α^ (Chen & Teixeira, 1986 ▸; Teixeira, 1988 ▸), but sometimes the exponent α changes between different values along *q* that are then usually covered by the Beaucage model (Beaucage, 1996 ▸). At this level, the connection of simple building blocks to form much larger structures is lost, and in turn form and structure factors are not revealed separately anymore. This multi-length-scale Beaucage approach stitches together several similar functions with individual radii of gyration *R*_g_ (indicating one prominent length scale) that all possess a Guinier range with a power law with individual exponent α towards higher *q*. The flattening of the Guinier range towards lower *q* describes a maximum size of aggregates/fractals, the internal structure of which no longer has any particular internal specificity.

However, porous materials may also display a preferred length scale on which repetitions occur (Schmidt, 1991 ▸; Walter *et al.*, 2003 ▸; Riedel *et al.*, 2023 ▸). This then results in a correlation peak that decays in a power law towards higher *q*, not necessarily with a universal power law exponent α. So far, no scattering model has included the description of a correlation peak with an additional power law with an arbitrary exponent α. The purpose of this article is to introduce such a model function. The combination (stitching together) of several peaked or non-peaked functions with specific length scales and specific exponents α can then be used to describe a wider range of structures within a generalized hierarchical multi-length-scale approach.

**Figure 1:**
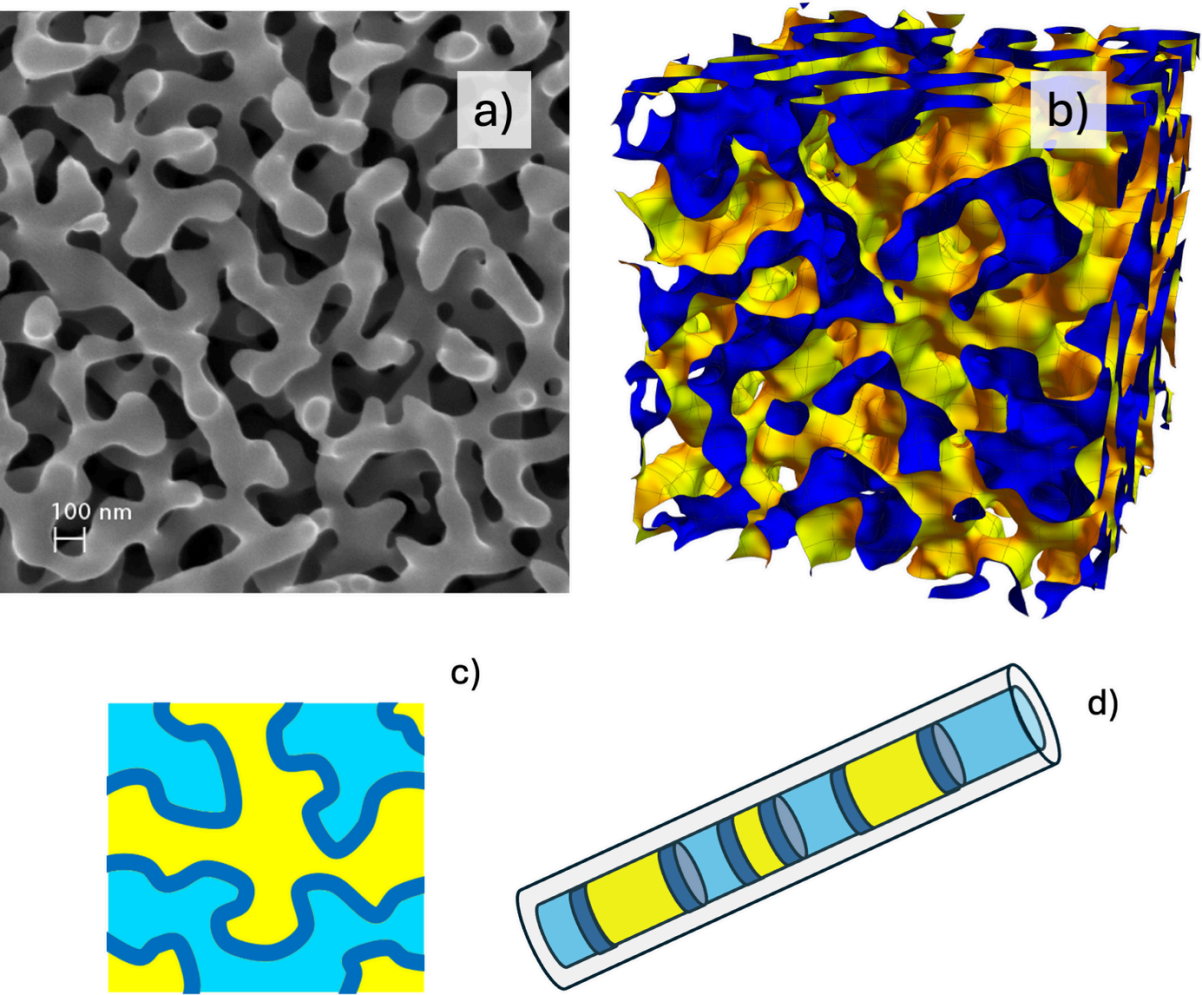
Examples of porous and bicontinuous structures from three to one dimensions. (*a*) Micrograph of porous glass provided by SCHOTT AG, reproduced with permission; https://www.schott.com/en-us/products/coralpor-p1000377. (*b*) A Gaussian random field simulation of a three-dimensional microemulsion. The surfactant film interface is displayed in yellow where it faces oil and blue where it faces water. (*c*) A two-dimensional porous structure. (*d*) A one-dimensional porous structure in a cylindrical pore. The surface versus bulk structure becomes clear by comparison of panels (*b*) with (*a*) and by the dark-blue portions in the structures displayed in panels (*c*) and (*d*).

Fig. 1 ▸ displays a visualization of single-stage porous and bicontinuous structures in one to three dimensions. The first example is shown through an illustrative micrograph of a porous glass provided by SCHOTT AG, included here to demonstrate a three-dimensional porous glass morphology. Similar glasses have been characterized using both imaging and scattering techniques (Schmidt, 1991 ▸; Walter *et al.*, 2003 ▸). Gold was also used to obtain such structures (Riedel *et al.*, 2023 ▸), with further samples that had a two-stage hierarchical structure. A Gaussian random field approach can be used to visualize three-dimensional microemulsions [Fig. 1 ▸(*b*)] (Pieruschka & Safran, 1993 ▸; Arleth *et al.*, 2001 ▸). The surfactant film interface is displayed in yellow where it faces oil and blue where it faces water. Here, it becomes clear that each domain of either oil or water is continuous in the whole space as a sponge structure where one domain hosts the other and vice versa. The corresponding structure in two dimensions is displayed in Fig. 1 ▸(*c*). This structure is not really bicontinuous anymore: that is possible in three dimensions only. This lack of continuity is even more evident in one dimension [Fig. 1 ▸(*d*)]; an example is provided by Prause *et al.* (2021 ▸).

A recent publication has reported that the coherent multiple scattering effect for *q* < 10^−4^ Å^−1^ may appear and result in surface instead of bulk scattering (Frielinghaus & Gommes, 2025 ▸). Examples with bulk and film structure can be seen in Figs. 1 ▸(*a*) and 1 ▸(*b*), respectively. The glass material fills a certain space and displays contrast against air. The displayed surface structure of a bicontinuous microemulsion is connected to the surfactant film and thus presents a surface structure in between oil and water. A coherent multiple scattering model also demands a model of the corresponding surface scattering with a, usually weaker, peak describing half the distance for repeating surfaces. So far, no model has related the bulk and surface scattering for the same porous material.

A rather sophisticated chord model (Levitz & Tchoubar, 1992 ▸) describes a first attempt at modeling porous materials using statistics for virtual chords of varying length present in the pore or the solid material (or the two domains). This can be obtained from real-space images with image analysis and then compared successfully with scattering curves. An analytical approach reported by Koberstein & Stein (1980 ▸) resulted in the Debye–Büche formula. In the approach adopted by Levitz & Tchoubar (1992 ▸), the need for two distinct distributions makes the formulation of a general model rather difficult. However, this generalization allows for correlation peaks in the scattering function with arbitrary peak width and intensity. As we will see below, the model reported here does not cover such a wide range of different peak parameters, but only a small number of parameters are needed.

The paper is structured in following way. Generalizations of the Teubner–Strey model for integer dimensions are derived in Section 2. This view neglects the original connection to the thermodynamic description and a new connection is made in Section 3. Expressions for fractional dimensions are derived in Section 4. After that, the embedding of lower dimensionalities into three-dimensional space is discussed in Section 5. In Section 6, the combination of several scattering functions to describe a hierarchical structure is shown. Finally, the paper is concluded in the last section.

## 2. Scattering functions of random porous media in one, two and three dimensions

**Figure 2:**
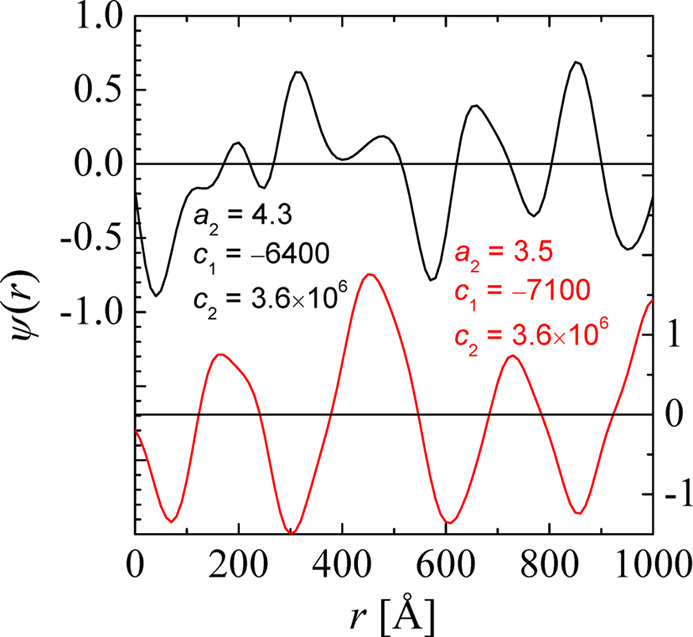
A line cut of the function ψ(*r*) for a three-dimensional microemulsion. Two sets of example parameters from equation (1), namely *a*_2_, *c*_1_ and *c*_2_, are given in units of thermal energy and the indicated length scales (Å). The top setting (black) corresponds to a correlation length ξ = 100 Å and the bottom setting (red) to ξ = 1000 Å. Positive values indicate the water domain and negative values the oil domain. The zeros are related to the positions of the surfactant molecules.

The scattering function derived by Teubner & Strey (1987 ▸) describes not only the scattering from bi­continous microemulsions but also approximately that from random porous media (Dahl *et al.*, 2024 ▸). In the latter case there remain slight deviations that can be explained by the technical process which is far from a thermodynamic equilibrium. However, the simple model grasps the essential properties of such a material quite well and assigns only two parameters, namely the domain spacing *d* and a correlation length ξ, to its structure. For microemulsions, the course of the formalism starts from the equilibrium Ginzburg–Landau free energy *F*, which considers the leading terms of a functional series (Chen *et al.*, 1990 ▸) where many non-symmetric terms are omitted:
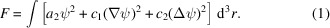
The fluctuating field ψ(*r*) takes values close to 1 for the water and close to −1 for the oil phase. The transition close to 0 is related to the surfactant that mediates between the water and the oil. In this simple approach, one only needs a single field ψ(*r*) (see Fig. 2 ▸), while, in general, a second field may extend the formalism to describe the surfactant properties in more detail (Theissen & Gompper, 1999 ▸; Arleth *et al.*, 2001 ▸). The first coefficient *a*_2_ is related to the thermodynamic miscibility of oil and water on large length scales. The mediating surfactant leaves the parameter *a*_2_ at slightly positive values. It also favours large interfaces between the water and oil domains and thus turns *c*_1_ to negative values. For reasons of stability, *c*_2_ must then be positive. From the fluctuation dissipation theorem one obtains the following macroscopic scattering cross section:
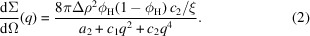
This is actually the denominator that follows from equation (1). When Fourier transforming equation (2) to real space, one identifies a correlation function γ_3_ with the two essential parameters:
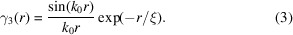
The domain spacing *d* is connected to a wavevector *k*_0_ = 2π/*d* that describes the alternating water and oil domains. The correlation length ξ describes a decay of strict repetitions with larger distances.

From all of that, the essential scattering function *B*_3_ is obtained (by connecting the coefficients *a*_2_, *c*_1_ and *c*_2_ to *k*_0_ and ξ) (Endo *et al.*, 2001 ▸): 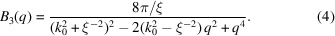
The missing calibration factor for small-angle scattering exper­iments [equation (2)] is (dΣ/dΩ)(*q*) = Δρ^2^ϕ_H_(1 − ϕ_H_)*B*_3_(*q*). It arises from consideration of a two-phase system (water and oil dominate the structure). Here Δρ is the scattering length density difference and thus describes the scattering contrast, and ϕ_H_ describes the volume fraction of hydrogenous material, which is seen as one of the two phases (it usually includes the surfactant), against the deuterated component.

In this formalism, a function 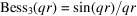 can be identified, which originates from the oscillatory part of the Fourier transform in *n* = 3 dimensions but reduces to the above-mentioned form for three-dimensional isotropic structures. The appearance of Bess_*n*_ is always tightly related to Bessel functions. It also appears in the oscillatory part of the correlation function 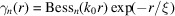 for *n* = 3. So the general relation between scattering function and correlation function is 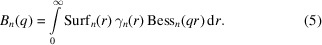
The surface term Surf_3_(*r*) = 4π*r*^2^ for *n* = 3 dimensions arises from the functional determinant of the isotropic integral in *n* dimensions. In the following, the correlation and scattering functions for *n* = 2 and 1 dimensions are explicitly derived, and the fluctuation dissipation theorem, which would result in identical scattering functions for all dimensionalities, is omitted. In three dimensions, the asymptote at high *q* corresponds correctly to the Porod law *q*^−4^, but it needs corrections for lower dimensionalities.

The second issue concerns surface scattering (film scattering for microemulsions), which is tightly connected to the bulk scattering from above. There are examples in the literature (Stephenson, 1966 ▸; Roux *et al.*, 1990 ▸; Roux *et al.*, 1992 ▸) where the real-space surface correlation function η_3_(*r*) is connected to the square of the bulk correlation function, *i.e.*
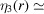

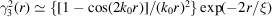
. In previous pub­lications (Frielinghaus & Gommes, 2025 ▸; Frielinghaus, 2026 ▸) it was finally concluded that the correct surface correlation function reads
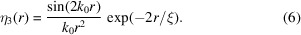
The oscillatory part needs to be corrected in the phase to have the origin of the correlations in the right place. Again, the corresponding scattering function is obtained by a Fourier transform:
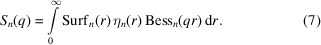
Explicitly, the surface scattering for *n* = 3 dimensions can be derived as
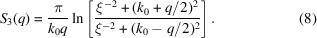
With the usual formalism for a two-phase system (with the surfactant one phase and the remainder the other phase), one obtains 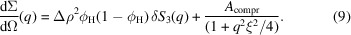
Here, δ is the surfactant film thickness. For microemulsions, a second term for the osmotic compressibility with amplitude *A*_compr_ is added. The reader is directed to earlier reports (Frielinghaus & Gommes, 2025 ▸; Frielinghaus, 2026 ▸) where the exact circumstances are discussed.

**Figure 3:**
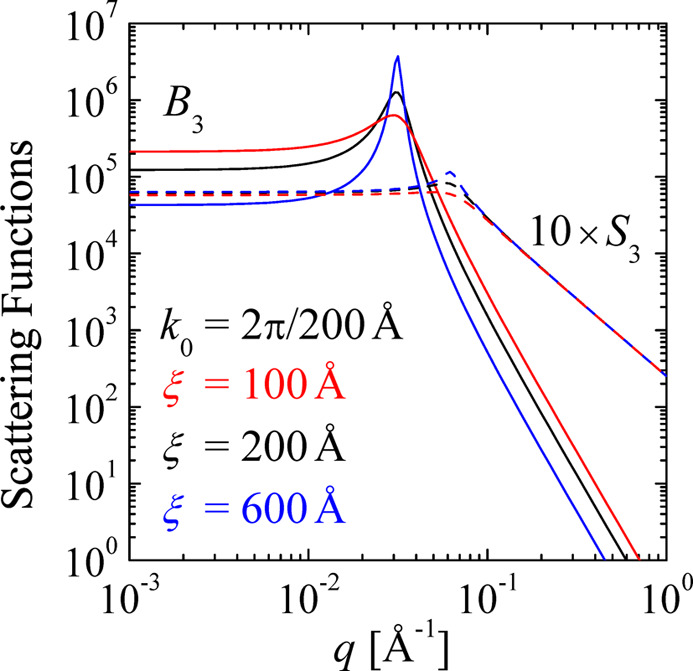
Example plots for a three-dimensional model of porous structures. The variation of the correlation length ξ from 100 to 600 Å is indicated.

To visualize the sensitivity of the bulk and surface scattering [equations (4) and (8)], a few examples are plotted in Fig. 3 ▸. The correlation length ξ varies from 100 to 600 Å. One can see that the bulk scattering is rather sensitive to ξ while the film scattering is not. This trend is maintained for all discussed dimensions *n*, as we will see below.

In the generalization to two dimensions, for the real-space correlation functions one arrives at
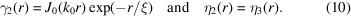
Here, *J*_0_(*qr*) is the Bessel function of zeroth order, which also describes the Fourier transform in two dimensions. The bulk correlation function strictly follows the above-mentioned recipes. However, one observes that the surface correlation function stays unaltered in two (and one) dimensions in order to obtain the correct high-*q* asymptotes.

The resulting integral can be simplified and connected to elliptical functions (Hanson & Puja, 1997 ▸; Kausel & Irfan Baig, 2012 ▸). This results in
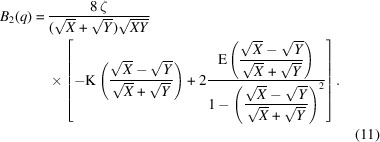
Here, the two elliptical functions are defined as
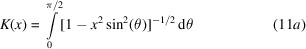
and 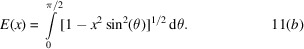
The arguments are abbreviated and one obtains ζ = ξ^−1^ and the two principal arguments *X* = (*k*_0_ + *q*)^2^ + ζ^2^ and *Y* = (*k*_0_ − *q*)^2^ + ζ^2^. For surface scattering, the result reads a little more simply when writing the 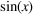 function as an exponential and collecting all exponentials together. The intermediate result relates to an arcsinh(*x*) function with a complex argument. The real-valued function can finally be written as
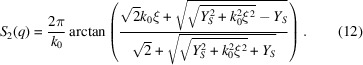
Now the abbreviation 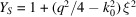 is defined. This exercise for the two-dimensional case is already mathematically demanding, although it gets easier again now. In one dimension the same principles as for two dimensions are obtained and one arrives at 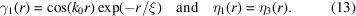
The Fourier transforms can be explicitly calculated now and one arrives at
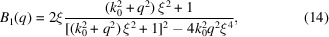

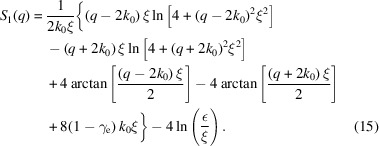
The latter equation results from an expansion in the variable (ε/ξ) where linear and higher orders are omitted. The parameter ε arises from the lower boundary of the integral in *r* and is tightly related to an atomistic size. I thus propose to set ε = 0.1 Å. The constant γ_e_ ≈ 0.577 is Euler’s constant. One-dimensional microemulsions have been experimentally investigated (Prause *et al.*, 2021 ▸) but with a slightly different interpretation.

**Figure 4:**
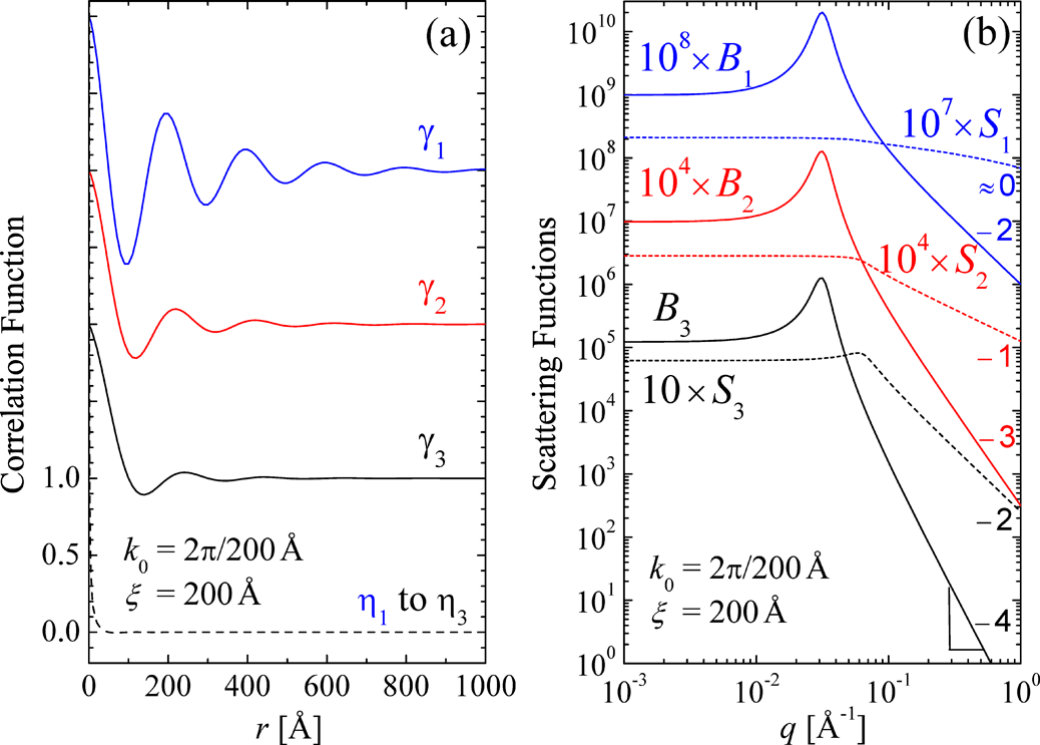
The generalized Teubner–Strey correlation and scattering functions for (*a*) bulk and film correlations γ_*n*_ and η_*n*_, and (*b*) bulk scattering *B*_*n*_ and surface scattering *S*_*n*_ in *n* dimensions. The spatial parameters *k*_0_ = 2π/200 Å and ξ = 200 Å are used. On a linear scale, the different correlation functions decay at different rates. However, all correlation functions are weighted by the factor Surf_*n*_ ≃ *r*^*n*−1^. At high *q*, the slopes of the double logarithmic scale of the scattering functions are indicated. The intensities on the vertical axis are based on different dimensions of space, so a comparison of absolute scales has to be made with care.

All correlation [equations (3), (6), (10) and (13)] and scattering functions [equations (4), (8), (11), (12), (14) and (15)] are plotted in Fig. 4 ▸. The structural parameters *d* = 200 Å and ξ = 200 Å are chosen here. The scattering functions *B*_*n*_ carry units of volume, surface and length according to the dimensionality *n* = 3 to *n* = 1. Thus, the units on the vertical axis do not really compare in terms of volumes and surfaces for the different dimensionalities. One observes for all bulk scattering functions a clear peak at around *q*_max_ ≃ 0.03 Å^−1^ and a much weaker peak or shoulder at *q* ≃ 0.06 Å^−1^ for the surface term. The bulk scattering asymptotically hits a power law of *q*^−1−*n*^ and the surface scattering a power law of *q*^1−*n*^. However, for one dimension, the latter is not represented exactly by a power law.

## 3. The link to the fluctuation dissipation theorem

**Figure 5:**
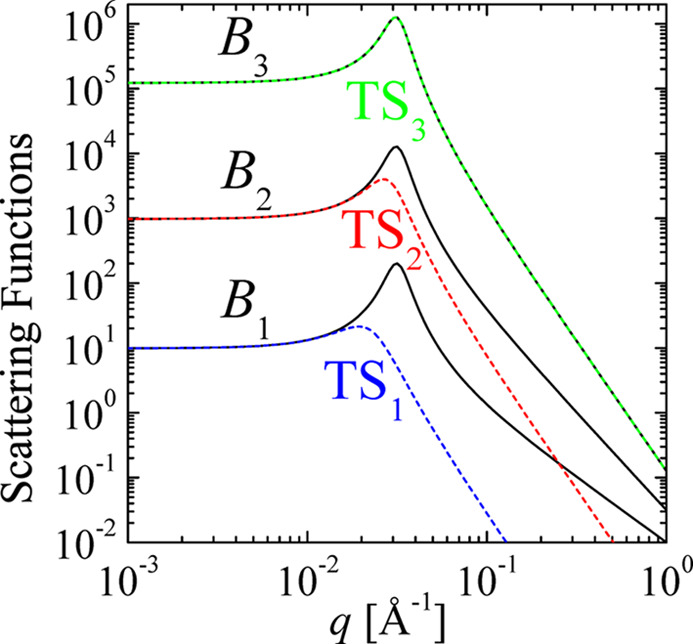
Bulk scattering functions (solid lines) with the corresponding Teubner–Strey expansions (dashed lines) in *n* = 3, 2 and 1 dimensions. The spatial parameters *k*_0_ = 2π/200 Å and ξ = 200 Å are used. The simple thermodynamic arguments have a much lower valid *q* range for the expansion at lower dimensions. This deviation gets worse the lower the dimension.

The unaltered real-space surface correlation function means that – in the ensemble average – the line cut is the same as that for correlating planes or volumes that can also be represented by multiple line cuts in parallel. However, the bulk correlation function carries a footprint of the dimensionality because the *n*-dimensional volumes stay connected, and so these functions look different for each *n*. One question remains: Why are only *n* = 3 dimensions ideal, where thermodynamic principles and reasons of correlation functions coincide? Does that mean that the fluctuation dissipation theorem is not valid in one and two dimensions? Or does it mean that the validity for small *q* is much more truncated in one and two dimensions? The latter case seems to be the most likely explanation, as discussed now. The resulting scattering functions of the Teubner–Strey type that are obtained from Taylor expansions of the correct scattering functions are plotted in Fig. 5 ▸, where the correct scattering functions are included for comparison. The deviations are larger for lower dimensions, and less representative coefficients (*a*_2_, *c*_1_, *c*_2_) produce well developed peaks. For the original three dimensions, there is of course no deviation between the two approaches. It is known that, for large scattering angles, microemulsions in three dimensions display more surface (Porod) scattering due to thermal fluctuations on small length scales (Frank *et al.*, 2007 ▸), but such arguments can also apply at all (or, better, *n* > 1) dimensions.

From the Taylor expansion of the reciprocal scattering function one obtains for one dimension 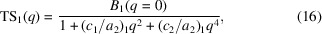

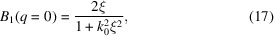

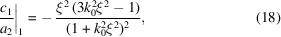

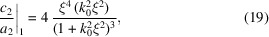

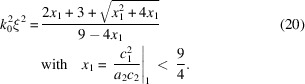
Equations (18) and (19) are solved for the respective coefficient ratios, while equation (20) is solved in the opposite direction to yield the product *k*_0_ξ. Then one only needs to solve equation (18) for ξ to obtain the individual spatial parameter. The remaining parameter *k*_0_ is then an issue of a simple division.

For two dimensions the same expansion can be made according to
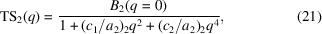

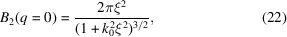

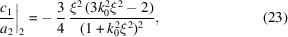

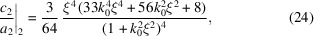

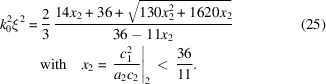
For completeness, the relations for the original Teubner-Strey theory are summarized as well: 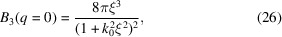

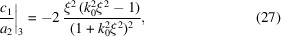

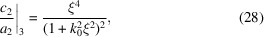

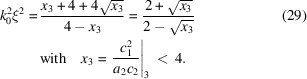


## 4. The generalization to fractional dimensions

The dimensionality of the scattering functions may take non-integer values. For this, the surface and Bessel-related functions are generalized according to
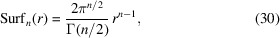

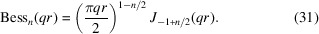


**Figure 6:**
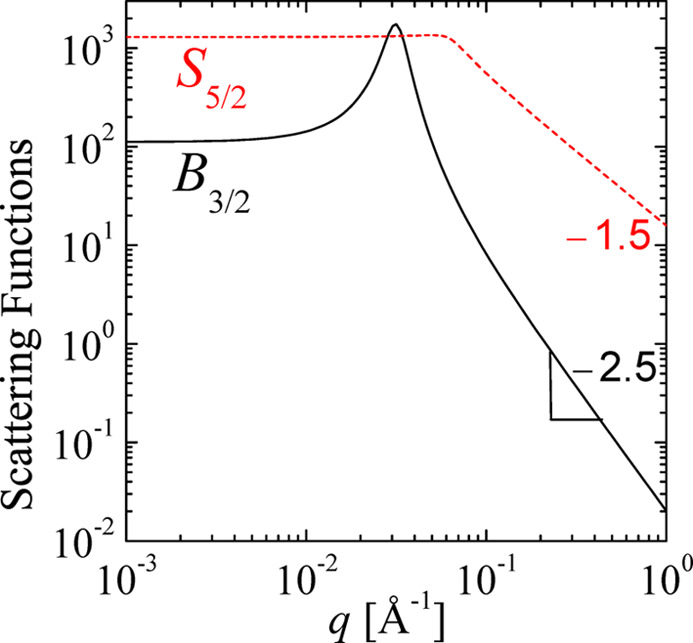
The generalized Teubner–Strey scattering functions for the bulk scattering *B*_3/2_ and for the surface scattering *S*_5/2_, which compare well in two dimension but have a deviating Porod scattering according to the parameter ν = 1/2. The spatial parameters *k*_0_ = 2π/200 Å and ξ = 200 Å are used. At high *q*, the slopes of this double logarithmic scale are indicated.

For the base dimension *n* = 2, one can consider the variations around the value *n* = 2 in the following sense. The bulk scattering function *B*_*n*−ν_ takes an asymptote at the high-*q* end with an exponent 3 − ν, while the surface scattering *S*_*n*+ν_ is represented by the exponent 1 + ν at the high-*q* end. The sum of the two exponents is 4 = 2*n*, which confirms the underlying space of the fractals. An example plot of the two scattering functions *B*_3/2_ and *S*_5/2_ (for ν = 1/2) is plotted in Fig. 6 ▸. There are also examples in the literature of deviations in fractal dimensions embedded in three dimensions (Walter *et al.*, 2003 ▸) (with an exponent of 3.6). One way to calculate the scattering functions is numerical integration in *r* space [equations (5) and (7)] (Van Deun & Cools, 2008 ▸). There are, however, relations with a certain hypergeometric function, known as an *R* function, that applies to our case of indices (Carlson, 1980 ▸), in contrast to one-dimensional infinite sums over hypergeometric functions that remain slow to compute (Reynolds, 2025 ▸). The *R* function can be further stripped to end up in a standard hypergeometric function: 
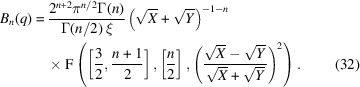
Here, 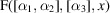 ≡ 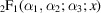 is the ordinary Gauss hypergeometric function. The abbreviations *X* and *Y* are defined below equations (11). Similarly, the surface scattering can be obtained (Olver, 2010 ▸; Olver *et al.*, 2025 ▸) as
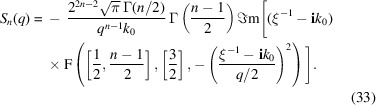
Here, **i** is the imaginary unit. The complex calculus is kept for brevity. For the exact integer numbers *n* = 1, 2, 3, equations (32) and (33) coincide with the simpler expressions from equations (4), (8), (11), (12), (14) and (15). Caution must be taken for the case *S*_*n*→1_, as already observed when discussing equation (15). There is no general recipe when *n* ≲ 1.1.

**Figure 7:**
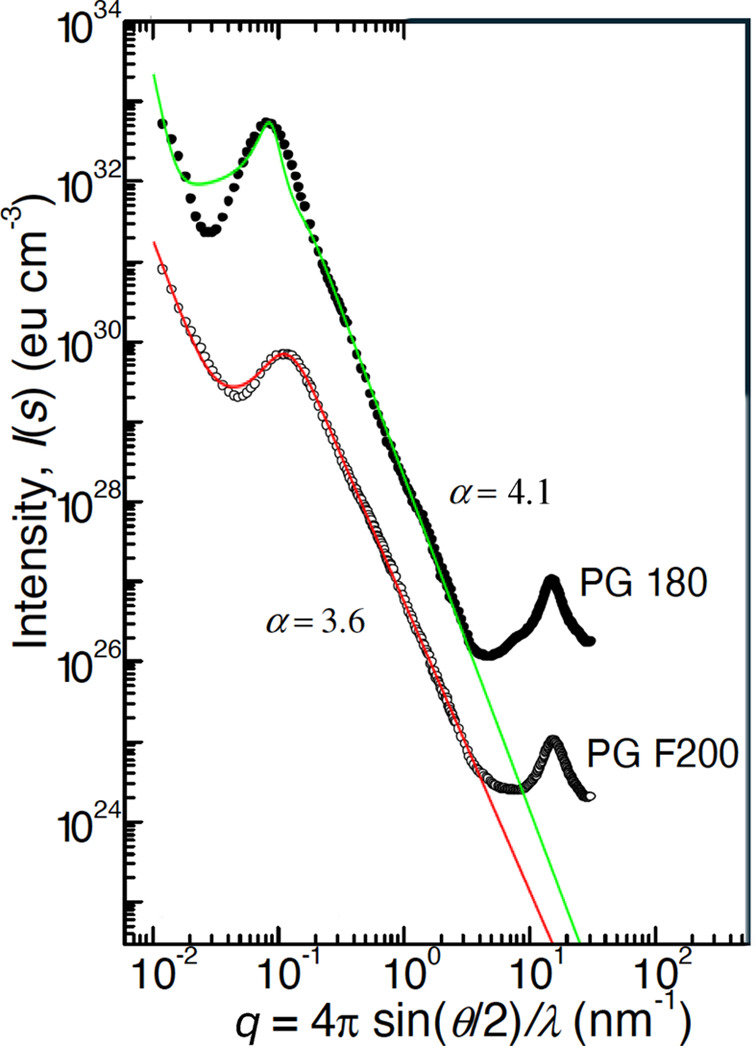
Two X-ray scattering curves taken from Walter *et al.* (2003 ▸) with the model function from equation (34). There is a power law at the smallest *q*, then a correlation peak at *q* ≃ 0.1 nm^−1^ and then a power law from the porous material over many length scales. Only the atomistic length scales are not covered by the current model. In this figure, the exponent α = 1 + *n* is explicitly given. The peak parameters of the different model curves are (green) *k*_0_ = 0.085 nm^−1^ and ξ = 60 nm, and (red) *k*_0_ = 0.108 nm^−1^ and ξ = 21 nm. Typical errors in the parameters are about ±10%.

For the bulk scattering of porous glasses, equation (32) must be slightly extended by the following terms: 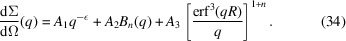
The first term describes a power law at the smallest *q* for a fractal on much larger length scales than the scattering experiment can reach. The second term describes the bare porous material. The third term comes in for slightly enlarged surfaces on smaller length scales than *d* and ξ. This has already been used for bicontinuous microemulsions (Frank *et al.*, 2007 ▸). The equations apply well to the porous glasses that were described by Walter *et al.* (2003 ▸), as we see in Fig. 7 ▸. All aspects of the pores are described well on many length scales. Only the atomistic length scales are omitted in the current theory.

As one particular case of equation (32) one can discuss the limit *k*_0_ → 0, which describes a case with no periodic structure. The hypergeometric function then disappears and a very simple formula is obtained,
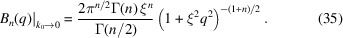
This is a generalization of the well known Debye–Büche formula (Koberstein & Stein, 1980 ▸; Levitz & Tchoubar, 1992 ▸) that can be directly compared with the Beaucage model function (Beaucage, 1996 ▸). Additionally, the formula contains an absolute amplitude that may facilitate the interpretation of fractal structures. On an absolute scale, the macroscopic cross section is finally given by (dΣ/dΩ)(*q*) = Δρ^2^ϕ_H_(1 − ϕ_H_)*t*^3−*n*^*B*_*n*_(*q*). The thickness *t* of the building blocks completes the formula for the correct units. Note that this modification of Ornstein–Zernicke-like scattering functions is often discussed in the context of critical phenomena (Fisher, 1964 ▸), albeit as an approach for small *q*. A combination of multiple equations (32) and (35) could describe multi-scale fractal structures over many length scales, similarly to the spirit of the Beaucage model. However, periodic structures would now be included in this description.

## 5. The complete structure including the missing dimensions

Let us go back to integer dimensions and exponents. When considering three-dimensional structures with two- or one-dimensional fluctuations, there remains an additional factor for the uncovered structure that completes the high-*q* power law to the expected exponent 4 or 2. This means a one-dimensional elongation results asymptotically in a *q*^−1^ factor and a two-dimensional plane in a *q*^−2^ factor. The more exact expression of the one-dimensional elongation with finite length *L* is 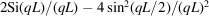 (Si is the sine integral function) (Pedersen, 1997 ▸). For a two-dimensional planar structure, one could chose a spherical surface with the factor 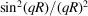. The factorization of two different scattering functions for different directions only holds when the length scales are separated. This means that *L*, *R* ≫ *d*, ξ, *i.e.* the structure is hierarchical, with large extensions in a rather ordered sense and fluctuations in the perpendicular directions on much smaller scales. An example with spherical symmetry might describe multilamellar vesicles, but here a much better model exists (Frielinghaus, 2007 ▸). However, the latest generalization to arbitrary dimensions now covers the description of porous materials with arbitrary fractal dimensions at the high-*q* end. At smaller *q* < *k*_0_ (in front of the correlation peak), the pure large-scale structure dominates the scattering curve.

## 6. An example of a multi-scale approach

**Figure 8:**
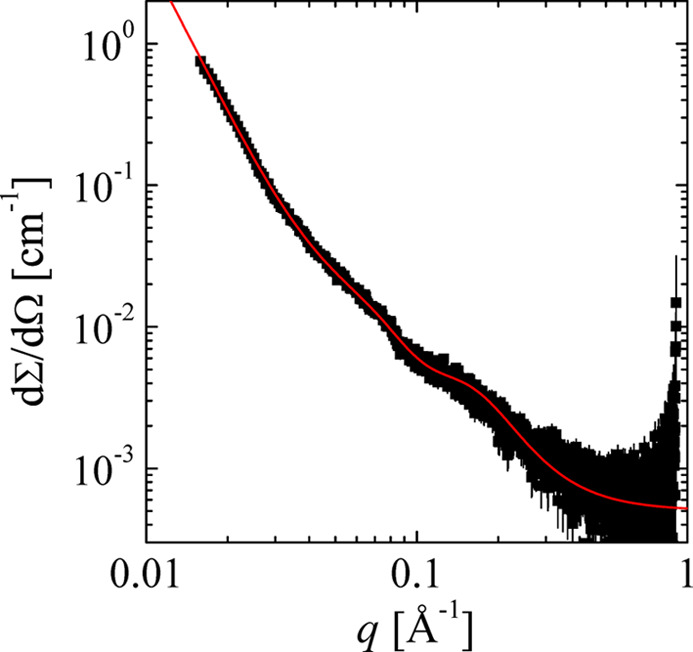
A SAXS scattering profile for an oat protein dispersion at 0.25% concentration that tends to aggregation. A multi-scale approach is used, as described in equation (36). The obtained parameters are *A*_1_ = 420 cm^−1^, ξ_1_ = 300 Å (out of the window), *n*_1_ = 3, *A*_2_ = 0.036 cm^−1^, ξ_2,0_ = 37 Å, *n*_2_ = 0.6, *k*_0,2,1_ = 0.07 Å^−1^, ξ_2,1_ = 24 Å, *A*_3_ = 0.0020 cm^−1^, *k*_0,3_ = 0.14 Å^−1^, ξ_3_ = 12 Å, *n*_3_ = 1.6 and *B*_gr_ = 0.00050 cm^−1^. Typical errors for all parameters are about ±10%.

Here, an example is given for stitching several functions together, as discussed above, to describe a rather complicated SAXS profile. The example is an oat protein dispersion that tends to aggregation. The scattering profile is depicted in Fig. 8 ▸. It is described by the following model function:
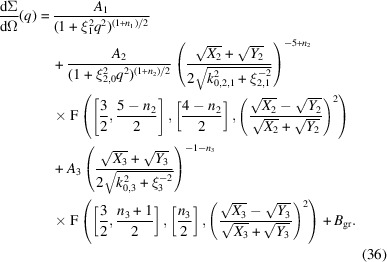
The abbreviations are 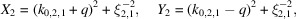

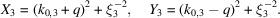
All contributions are scaled such that the amplitudes *A*_*i*_ are given in absolute units (cm^−1^) as a virtual forward scattering. Note that the second term, with *A*_2_, contains a simpler power law with a rather shallow slope for smaller *q*, which is then multiplied by a peaked function. The high-*q* exponent of this term is constrained to α = 6. This whole description illustrates the possibilities for stitching several simpler scattering laws in the form of equations (32) and (35) together to obtain a multi-length-scale model.

## 7. Conclusions

With the work presented here, a complete toolbox for porous materials with arbitrary dimensionality is now available. It describes a correlation peak for alternating domains and a power law at higher *q*. The scattering function may be related to thermodynamic parameters in the case of microemulsions of arbitrary dimensionality. However, the connection to thermo­dynamics at lower dimensions seems to be rather weak.

The whole new toolbox broadens the applicability of porous and/or bicontinuous models to many more scattering experiments than before. A more universal multi-length-scale approach is obtained by stitching multiple peaked or non-peaked model functions together, and this could replace the widely accepted Beaucage model.

## References

[bb1] Arleth, L., Mar˘celja, S. & Zemb, T. (2001). *J. Chem. Phys.* **115**, 3923–3936.

[bb2] Barker, J. G., Glinka, C. J., Moyer, J. J., Kim, M. H., Drews, A. R. & Agamalian, M. (2005). *J. Appl. Cryst.* **38**, 1004–1011.

[bb3] Beaucage, G. (1996). *J. Appl. Cryst.* **29**, 134–146.

[bb4] Carlson, B. C. (1980). *SIAM J. Math. Anal.* **11**, 428–435.

[bb5] Chen, K., Jayaprakash, C., Pandit, R. & Wenzel, W. (1990). *Phys. Rev. Lett.* **65**, 2736–2739. 10.1103/PhysRevLett.65.2736 10042679

[bb6] Chen, S.-H. & Teixeira, J. (1986). *Phys. Rev. Lett.* **57**, 2583–2586. 10.1103/PhysRevLett.57.2583 10033804

[bb7] Dahl, M., Gommes, C. J., Haverkamp, R., Wood, K., Prévost, S., Schröer, P., Omasta, T., Stank, T. J., Hellweg, T. & Wellert, S. (2024). *RSC Adv.* **14**, 28272–28284. 10.1039/d4ra04090b PMC11372560 39239284

[bb8] Endo, H., Mihailescu, M., Monkenbusch, M., Allgaier, J., Gompper, G., Richter, D., Jakobs, B., Sottmann, T., Strey, R. & Grillo, I. (2001). *J. Chem. Phys.* **115**, 580–600.

[bb9] Fisher, M. E. (1964). *J. Math. Phys.* **5**, 944–962.

[bb10] Frank, C., Frielinghaus, H., Allgaier, J. & Prast, H. (2007). *Langmuir* **23**, 6526–6535. 10.1021/la0637115 17489617

[bb11] Frielinghaus, H. (2007). *Phys. Rev. E* **76**, 051603. 10.1103/PhysRevE.76.051603 18233665

[bb12] Frielinghaus, H. (2026). *J. Appl. Cryst.* **59**, https://doi.org/10.1107/S1600576726005066. 10.1107/S1600576726005066 PMC13440040 42559285

[bb13] Frielinghaus, H. & Gommes, C. J. (2025). *J. Appl. Cryst.* **58**, 1553–1570. 10.1107/S1600576725006685 PMC12502871 41064418

[bb14] Hanson, M. T. & Puja, I. W. (1997). *Q. Appl. Math.* **55**, 505–524.

[bb15] Ji, Y., Radlinski, A. P., Blach, T., de Campo, L., Vu, P., Roshan, H. & Regenauer-Lieb, K. (2022). *Fuel* **325**, 124957.

[bb16] Kausel, E. & Irfan Baig, M. (2012). *Q. Appl. Math.* **70**, 77–97.

[bb17] Koberstein, J. T. & Stein, R. S. (1980). *J. Polym. Sci. Polym. Phys. Ed.* **18**, 199–205.

[bb22] Levitz, P. & Tchoubar, D. (1992). *J. Phys. I Fr.* **2**, 771–790.

[bb18] Magerl, A., Lemmel, H., Appel, M., Weisser, M., Kretzer, U. & Zobel, M. (2024). *J. Appl. Cryst.* **57**, 1282–1287. 10.1107/S1600576724007246 PMC11460398 39387068

[bb19] Nishida, K., Ogawa, H., Matsuba, G., Konishi, T. & Kanaya, T. (2008). *J. Appl. Cryst.* **41**, 723–728.

[bb20] Olver, F. W. (2010). *NIST Handbook of Mathematical Functions.* Hardback and CD-ROM. Cambridge University Press.

[bb21] Olver, F. W. J., Lozier, D. W., Boisvert, R. F. & Clark, C. W. (2025). *NIST Digital Library of Mathematical Functions.*https://dlmf.nist.gov.

[bb23] Pedersen, J. S. (1997). *Adv. Colloid Interface Sci.* **70**, 171–210.

[bb24] Pieruschka, P. & Safran, S. A. (1993). *Europhys. Lett.* **22**, 625–630.

[bb25] Pilz, I., Glatter, O. & Kratky, O. (1979). *Methods Enzymol.* **61**, 148–249. 10.1016/0076-6879(79)61013-3 481226

[bb26] Prause, A., Hörmann, A., Cristiglio, V., Smales, G. J., Thünemann, A. F., Gradzielski, M. & Findenegg, G. H. (2021). *Mol. Phys.* **119**, e1913255.

[bb27] Reynolds, R. (2025). *arXiv*, 2502.14876.

[bb28] Riedel, L., Markmann, J., Weissmüller, J. & Shi, S. (2023). *Phys. Rev. Mater.* **7**, 116001.

[bb29] Roux, D., Cates, M. E., Olsson, U., Ball, R. C., Nallet, F. & Bellocq, A. M. (1990). *Europhys. Lett.* **11**, 229–234.

[bb30] Roux, D., Coulon, C. & Cates, M. E. (1992). *J. Phys. Chem.* **96**, 4174–4187.

[bb31] Schelten, J. & Schmatz, W. (1980). *J. Appl. Cryst.* **13**, 385–390.

[bb32] Schmidt, P. W. (1991). *J. Appl. Cryst.* **24**, 414–435.

[bb33] Stephenson, J. (1966). *J. Math. Phys.* **7**, 1123–1132.

[bb34] Teixeira, J. (1988). *J. Appl. Cryst.* **21**, 781–785.

[bb35] Teubner, M. & Strey, R. (1987). *J. Chem. Phys.* **87**, 3195–3200.

[bb36] Theissen, O. & Gompper, G. (1999). *Eur. Phys. J. B* **11**, 91–100.

[bb37] Van Deun, J. & Cools, R. (2008). *Comput. Phys. Commun.* **178**, 578–590.

[bb38] Walter, G., Kranold, R., Enke, D. & Goerigk, G. (2003). *J. Appl. Cryst.* **36**, 592–596.

[bb39] Zhang, F. & Ilavsky, J. (2010). *Polym. Rev.* **50**, 59–90.

